# Impact of COVID-19 on the lives and mental health of children and adolescents

**DOI:** 10.3389/fpubh.2022.925213

**Published:** 2022-10-18

**Authors:** Fengxiao Li

**Affiliations:** School of Management, Hangzhou Dianzi University, Hangzhou, China

**Keywords:** COVID-19, higher education, social life, anxiety, lockdown

## Abstract

Student mental health is an integral part of the fight against disease, and health was an evolving concern during the spread of COVID-19. As the COVID-19 pandemic unfolds, physical distancing and social restrictions were introduced, and because of these, it was found a great impact on students' lives and their mental health. Adolescent mental health is focused on prevention, early detection, therapeutic innovation, and service development. In these circumstances, service providers can expand telemedicine Digital services that may help provide future mental health services to young people, particularly students. This study collects and analyzes data from students on the impact of these new online learning techniques, and by collecting and analyzing the challenges and issues faced by college students during the COVID-19 pandemic. The questionnaire was based on and extended the European Students' Union Survey and targeted higher education students concerning what student life looked like during the pandemic, including teaching and learning, their social contacts, habits/routines, as well as how they were coping with the situation emotionally and financially. The case of universities and distance learning education units in higher education during the COVID-19 pandemic process. This article highlighted the impact of COVID-19 on students of all ages and their time schedules such as online learning and reflection, study environment, sleep habits, routines, and outcomes. It was found that the students generally require greater self-discipline and motivation to complete online classes. It was also reflected that the pandemic adversely affected student mental health, leading to an increased prevalence of Major Depressive Disorder (MDD) and Generalized Anxiety Disorder (GAD). This may have a significant impact on their mental health issues such as frustration, stress, and sadness. This will significantly lead to cooperation between various local authorities and the private sector.

## Introduction

Psychosocially, COVID-19 disproportionately affects young people. The stresses and restrictions make college students more likely to develop mental health problems that may affect their academic success, social intelligence, future careers, and personal opportunities. Both short-term and long-term factors affecting young people were social isolation, changes in the provision of treatment services, and the near-total loss of all structured professions (e.g., School, work, education). The World Health Organization declared COVID-19 a global pandemic, forcing many higher education institutions to take steps to promote student safety. Although there is no systematic review of the effects of COVID-19 on mental health, however some studies related to pandemics (including bird flu and SARS) have shown adverse effects on the mental health of affected people. Therefore, the development of COVID-19 was predicted to cause general psychological reactions such as tension, fear, anxiety, and mental disorders such as acute stress disorder, post-traumatic stress disorder, depression, and suicide. Interestingly, students who reported at least one effect showed a higher level of resilience and a self-reported health score, but with less need for support. Concerning exercise, those who exercised constantly had lower levels of depression and anxiety, higher quality of life, and higher self-assessed health scores than those who did not exercise occasionally (1–3).

Liquor utilization was not related to any of the choice factors, but tobacco utilization was altogether related to five choice factors, appearing to be higher uneasiness and misery, lower life quality of life, self-reported well-being, and higher back needs (4). These factors were significant impacts on children's health and well-being. The educational network takes serious efforts to seriously preserve the continuity of training during this period, while children and universities were to rely on their resources to study remotely through the internet, television, or radio. Teachers had to adapt to a new educational concept and delivery method so that they would not be able to do anything more than new educational concepts (5). This disaster revealed several disadvantages and disadvantages from whether the broadband and computer systems needed to support online education as well as the environment necessary to change the availability, assets, and requirements of computer systems.

Traditional education was violated due to the clogging of COVID-19 (6). Despite the considerable efforts of the education network to maintain the continuity of training for this period, college children and students were to rely on resources to learn remotely through the Internet, television, or radio. Teachers were to adapt to new pedagogical ideas and teaching methods they were never taught before. Newcomers from underserved group lack resilience and motivation to learn, as well as access to virtual learning resources. The universities gradually return to their previous educational levels, and the slow rise of today's college students' skills shortages will only be seen in the long run. However, it is important when considering the impact of this period.

In other words, if the educational system is recovered before the productivity preparation, the state will continue to fight low socioeconomic welfare. Some groups may be more vulnerable to the psychosocial effects of the COVID-19 pandemic than others (7–9). Because they were in a critical period of development, with half of all mental health disorders developing before the age of children and adolescents must be provided with adequate support. Factors associated with mitigation measures such as social distancing, family discord, school closures, fears about the future, and quarantine are disrupting young people's lives (10, 11).

These disruptions include changes in routine, a break in the continuity of learning with the closure of schools, a break in health care missed significant life events, and the loss of a sense of security and safety.

It was examined that the student's psychological well-being and well-being in higher education are among the broader predictors of COVID-19 (12, 13). Perhaps the most striking finding was that levels of distress and discomfort were high, with more than half of the students exceeding clinical limits. This suggests that they are likely to be in clinically significant distress and/or discomfort at the time of the study.

Despite the rapid replacement of the lecture on the facial lectures of online learning, such closure was influenced by training and testing and experienced international security students, international security students, and criminal reputation in host countries. In addition to academic content, this case is most concerned about the value of college education with network and social opportunities. If digitization is to grow and deepen relationships between students, coaches, and others, universities will need to rethink the learning environment. The addition of positive cases once again shows that there are still not yet been isolated, that there is still an infection due to close contact, and that no one is conscientious about washing hands with soap. Citizens are responsible for implementing various government-issued guidelines to break the COVID-19 infection chain, such as staying at home, maintaining a physical distance of at least 1 m, and washing hands with soap. It is a great need to exercise discipline such as the policy of the Ministry of Health of Indonesia applies to public health (14, 15).

The COVID-19 pandemic affects people psychologically; because the spread of it continues, death rates increase day by day, life comes to a halt and its control time is unpredictable. Similar to the traumatic experiences that have caused many psychological problems in humans throughout history, COVID-19 will have a negative psychological effect on every person. Scientific research on COVID-19 is of great importance to prevent its destructiveness of it in social, political, psychological, educational, and economic dimensions. After the COVID-19 epidemic is controlled over time, the psychological effects on people will be clearer. It is estimated that its effect will cause many problems in the short and long term (16–18).

In this study, the possible psychological effects of COVID-19 on children, youth, elderly people, and healthcare staff were discussed considering theoretical information (19, 20). To minimize negative psychological effects, some recommendations were also presented. Since the government caused the spread of illness, the sanitary crisis is a major financial disaster that is expected to load society for many years because the hygiene crisis is deactivated the financial system.

According to the Current Economic Prediction of the OECD, even the most optimistic scenarios, forecasts a harsh economic downturn. According to the OECD, even if secondary infections are avoided, global economic activity is expected to contract by 6% in 2020. According to the statistics in Table 1, universities have conducted effective and successful educational courses during the pandemic. However, it is very important to consider the current location of distance learning students. The purpose of this study was to determine students' knowledge and views on distance learning between education and training pandemics in the spring semester of 2019–2020.

**Table 1:** The distance education learning statistics performed in the 2019–2020 spring pandemic.

	**Data**
The total number of used equipment	13.244
Online lectures	2.985
live class participation of students	35.620
Number of accesses to asynchronous resources	171.372
Number of attendances during lectures	34.350
Watched live lecture	73.308
Number of teachers and staff	855
Number of students included in the distance education system	27.039
Uploaded visual program size	180 GB
Total number of files upload size	214 GB

In this case, the most important thing is learning must occur even at home. Without teachers, the target must be that the curriculum must be achieved. Not move the school at home but choose essential materials that children need to do at home. Second, the teaching staff or teacher must provide education to children about life skills, namely education that is contextual following the conditions of each home, especially the understanding of COVID-19, regarding the characteristics, how to avoid it, and how to prevent someone from contracting (21).

On the other hand, government investments are often violated in response to external shocks because the government requests investment. The slowdown in economic growth caused by the spread of viruses also affects public investment in OECD and colleagues' education, and tax income of autumn and emergency and emergency budgets is aimed at helping health spending and welfare (22–24).

**Figure 1:**
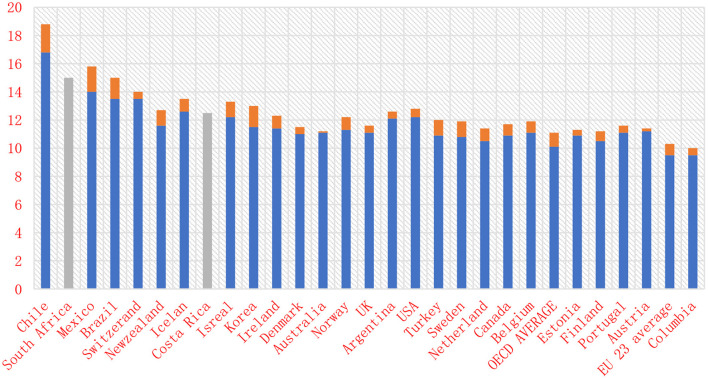
Total public expenditure on education as a percentage of total government expenditure (2017).

Public investment in education should not be encouraged by multiple members, as there is no guarantee that the market will provide fair access to education. In 2017, public spending on education accounted for 11% of total costs in OECD countries. However, according to data from the OECD and related countries, this ratio fluctuates between about 7% and about 17% in Greece (Figure 1).

The previous economic crisis put a heavy burden on the national budget. This reduced public spending on education in some countries. Cross-country and other comparisons do not show a strong link between education spending and academic performance across European countries, but the overall school system performance is so different that spending without improving grades. Reducing the pleasure of learning and the risk of injury. It can also take time to assess how disasters affect education spending. The previous economic crisis put a heavy burden on the national budget (25–27).

As a result, the cost of public education has fallen in some countries. The performance of the education system does not change much because the performance of the education system is very different. Therefore, there is no need to undermine the strong relationship between counterpoint and academic performance. It can take some time to assess how depreciation affects training costs. Despite severe fiscal cuts in all OECD countries since the last economic disaster, most OECD countries increased government spending on education between 2008 and 2009 and 2010, with 2010 being the most effective (28).

In April 2020, the support package for the highest level of education was presented. As a result of the investment in the Australian government, this package reduces the cost of short online processes and is not only released from mortgages in the house of young people that began in May but as well as investment for home college students (29). International students were particularly affected early in the blockade because they struggled with how the closure of the university affected their reputation on campus and in the host country. Students were to choose between returning with little information about when they could return or staying in a host country with limited employment and education opportunities while waiting for a visa to be issued. In some countries, such as Canada and the United Kingdom, students are exempt from visa requirements and are allowed to stay on campus, but this is no longer the case everywhere. Higher education institutions are using technology to offer online courses and student ratings as an elegant alternative to ensuring continued education despite the blockade (30, 31).

Before the pandemic, many higher education institutions made online guides available, but few students saw this as the only way to physically characterize. The countries that rely heavily on international college students to pay various fees, such as Australia, Canada, the United Kingdom, and the United States, will be hit hardest. For example, in 2017/18, public universities in Australia, Canada, and the United States charged international students more than the US $ 13,900 per year at the same level as undergraduate or domestic students.

**Figure 2:**
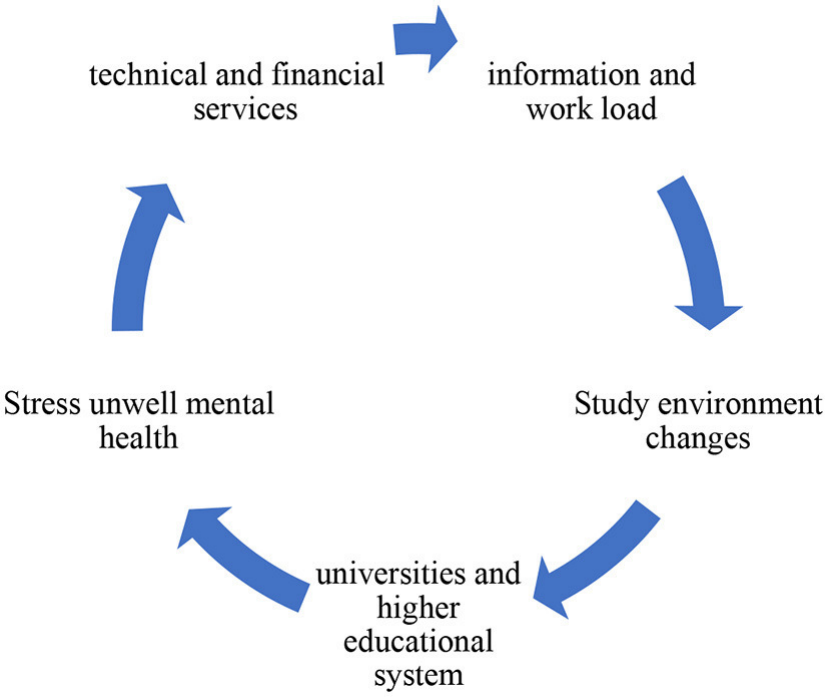
Summary of challenges due to the COVID-19 pandemic on university students.

The three main challenges are related to technical problems and online learning challenges as summarized in Figure 2:

(a) Information and work overload.
(b) Difficulty adapting and unfamiliarity with the new online learning environment.
(c) Personal health challenges related to stress and anxiety problems.

## Materials and methods

The main goal of this paper is to highlight the major results of a global survey on the impacts of the COVID-19 pandemic on the life of higher education students that was carried out by an international consortium of universities, other higher education institutions, and students' associations, and what they were expecting by way of support measures from various institutions, e.g., universities, the government, banks, etc. (32, 33). To understand the ways that the COVID-19 pandemic has impacted a range of aspects of student lives, the following research questions were addressed:

A1: How have students around the world been satisfied with different aspects and elements of student life during the COVID-19 pandemic and how have they perceived them?
A2: Are there any socio-demographic and geographic differences in:

- Students' satisfaction with and perception of selected elements of academic work and academic life due to the transition from onsite to online lectures.
- Students' perception of the COVID-19 pandemic's consequences on their social and emotional life, personal circumstances, and habits.
- students' satisfaction with the role of selected institutions and their measures during the COVID-19 pandemic.

This study will lead to focus on the COVID-19 Pandemic in the lives of Students and their intellectual health through the demographic information of the participant.

The method of investigation is defined as “precautionary measures taken on the entire universe, or on a sample or group of samples taken from it to reach a general judgment about the universe.” During the COVID-19 pandemic, survey methods were used to gather student opinions on distance learning (34).

According to the data, the socio-demographic and other characteristics of the study population are shown in Table 1. Approximately two-thirds of the sample of 40,385 higher education students (75%) and more than half of children (55%) of the population fall in the age range of 10–15 and number are 350 and a total of 30% are affected. Most of the respondents were domestic (95%), full-time (88.1%), and first level (81%) students. A little over one-third of the participants (37.0%) were studying social sciences, followed by applied sciences (31.1%) and natural and life sciences (21.7%). A scholarship was not held by 70.8% of the respondents in 2019/2020 and just over half of them (52.6%) were able to pay the overall costs of their study before the COVID-19 pandemic (11).

During the 2019-2020 pandemic, the research was conducted with the support of university students. Participants were selected based on the principles of autonomy and availability. The survey received 1.10 responses, of which 712 boys (70.5%) and 399 girls and women (39.5%) participated. As shown in Table 2.

**Table 2:** Demographic information of the participants.

**Gender**	**Participation**	**Percentage (%)**
Under age 0–15	350	30%
Number of male	712	70%
Number of female	399	39%
**Device used in distance learning education**		
Computer	566	56%
Phones	530	55%
Tablets	13	13
Urban location	8/10	8%
**Computer and social status**		
Yes	661	61.5
No	490	49.5
**Internet connection except for telephone**		
Yes	636	63.5
No	440	40.5

When asked which device they prefer for distance learning, participants found that they used 566 computers (56.1%) and 530 mobile phones (55.4%) as devices. “Even if you have access to the distance learning system over the phone, do you have a computer that you can use individually?” 551 (54.5%) responded positively.

The analysis moves from descriptive to interpretive. The process consists of first categorizing each transcript into broad themes and through continued review of the data into more specific data about students. The Public Health and the subjective lived experiences of people, and therefore, the researchers demonstrate reflexivity and the behavior changes effects and Depression/Anxiety among young people throughout the study (see Table 3).

**Table 3:** The referral and percentage of qualitative data.

**Subjective**	**Referral out of 48**	**Percentage**
Children appearance	25	55%
Social isolation	36	80%
Stress over home-schooling	32	69%
Behavioral changes	18	35%
Difficulty being confined in the household	21	48%
Depression/anxiety among young people	14	29%

To elaborate, reflexivity encourages the researchers to consider how their subjective worldviews may impact the research process. This approach benefits this research's quality, particularly concerning the data analysis as it buffers against personal experiences and biases impacting the research findings.

The data about the “Distance Education System,” “Lecture / Lecture Notes,” and “Individual Evaluation,” was designed to explore the student's perspective on distance education. The scale is created using a 5-point Likert scale and has a Cronbach's alpha reliability factor of 0.909.

The method of Means, standard deviations, and tests were calculated using the SPSS (Statistical Package for the Social Sciences) program. Responses to scale items were evaluated using mean, standard deviation, frequency, percentage, and *t*-test analysis. The 5-point Likert scale is a rating scale ranging from 1 to 5. As shown in Table 4.

**Table 4:** Scale options and score ranges.

**Options**	**Range**	**Score range**
Completely agree	5	4.30–5.00
Agree	4	3.50–4.20
Not sure	3	2.70–3.30
Not sure	2	1.80–2.50
Refuse	1	1.00–1.99

In a crisis such as the COVID-19 pandemic, many questions emerge, and students need the support of various services. The survey results show that the students, regardless of the continent, studying from home commonly require greater self-discipline and motivation to follow through with online lessons, particularly in the earlier period when students were getting used to the new system, which might affect their feeling of an increase in study obligations. On the other hand, lecturers unfamiliar with the new mode of delivery could overload their students with study materials and assignments. Therefore, the students were asked to compare their workload before the onsite classes were canceled with the new circumstances after the lockdown (35).

## Results and discussion

When the world tackled the outbreak of the COVID-19 pandemic, higher education was a significant impact on its core students. For them, time was undoubtedly unprecedented and extremely stressful, with face-to-face events being moved online, semesters postponed, and exams being coordinated. Therefore, there is an urgent need for detailed research on how the pandemic crisis affected the lives of students around the world. After providing a comprehensive review of the current literature, our treatise is the first large-scale global survey of students from a variety of research perspectives since the outbreak of COVID-19 (36). This study was conducted between 5 May and 15 June 2020 and found that student life during the COVID-19 pandemic was academically, socially, emotionally, economically, and otherwise. The author tried to explain what it was like. In this regard, it provides valuable and unique detailed insights into student life during the blockade (World Health Organization).

The 21-item survey conducted as part of this survey was represented by three elements: “Distance Education Module,” “Lectures and Lecture Notes,” and “Individual Evaluation,” and the results are shown under these headings. In addition, a test analysis of the participant's computer and internet conditions was performed. In the first attempt to stop the spread of the virus, many countries-imposed blockades, and closed faculties and/or universities in OECD and partner countries for several months. In response to the COVID-19 pandemic, the People's Republic of China was the first of the 38 educated countries and other partner countries to close their faculties in 2020. On 16 February 2020, school closures were imposed in some parts of China when the planned spring semester began early and was extended nationwide about a week later. As the pandemic spread, other countries began to close their faculties (final school buildings without completely closing coaching and learning). Preliminary data from various sources (refer below) show how people react during this ongoing and evolving pandemic. By the end of March 2020, all 46 educated countries had immediate school closures of varying degrees. The United States and other 41 countries have closed their schools and five (Australia, Iceland, the Russian Federation, Sweden, and the United States) as can be seen in Figure 2. However, not all pandemic-affected countries are forced to close all schools. For example, if the elegant size is <20, it will continue to be available to the top teachers in Icelandic.

Most elementary and junior high schools in Sweden remained open, but high schools switched to distance learning in mid-March (UNESCO, 2020). It is difficult to estimate the number of weeks of education affected in each country, as individual faculties and local governments in some countries have autonomy over time.

By the end of June 2020, grade and school reopening employers will have several diplomas in two countries (4%) for up to 7 weeks, six countries (13%) for 812 weeks, and 12–16 weeks enacted, 1618 weeks (28%) in 24 countries (52%), 1618 weeks (28%) in 13 countries, and 18 weeks or more in China (UNESCO, 2020).

First, the student's academic work and academic life aspects were studied. Due to the physical closure of higher education institutions, the majority of teaching and learning processes went online, i.e., 89% of all respondents claimed that their onsite classes were canceled and substituted with online lectures in the form of real-time video conferences, sending presentations to students, video recordings, and written communication (forums and chats) (37).

**Figure 3:**
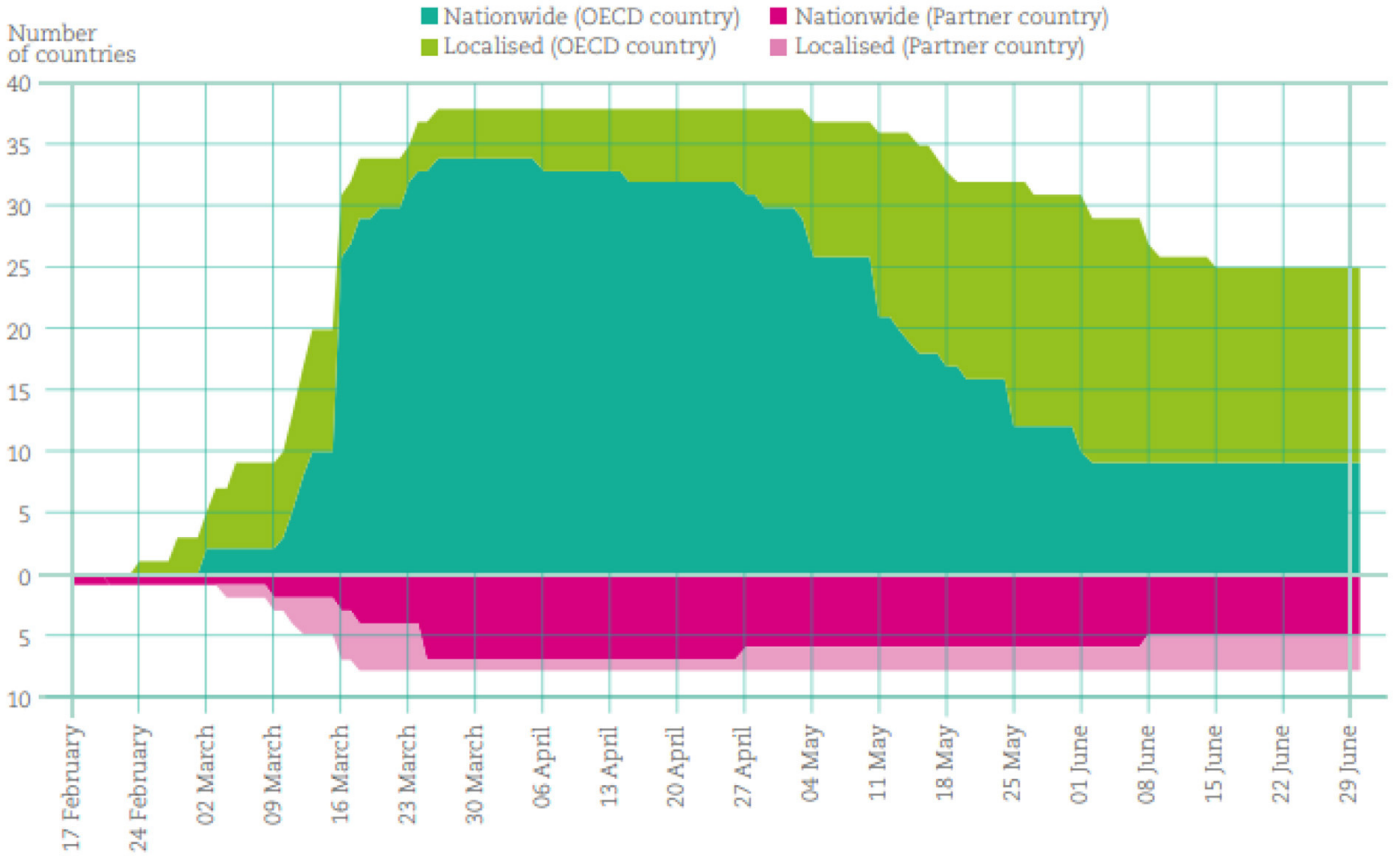
Number of countries with school closures due to COVID-19 Data covers the period between 17 February 2020 and 30 June 2020 (UNESCO, 2020).

The overall effect may not be so dramatic, as some of these times coincided with the planned faculty leave. Easter and/or spring break in April and early May reduced the impact of teacher closures by up to 2 weeks in many countries in Europe and the Southern Hemisphere. For example, in Japan, there was a 2-week spring break at the end of March refer to Figure 3 (25, 38). In addition, some countries reorganized their academic year to make up for the lack of coaching time. For example, in some jurisdictions of Australia and Chile, cold teacher holidays were carried forward. The summer season was shortened in South Korea.

### Measures to continue students learning during school closure

Countries used a variety of resources to guide students‘ learning when they were unable to return to school. In the OECD and partner countries, online platforms were the most prominent tool used during school closures (39). Their homeschooling was available to all college students in elementary and junior high school (national level of the Ministry of Education). Greek teachers used real-time digital education with a variety of online learning tools (Ministry of Education). As shown in Table 5.

**Table 5:** Universities and all higher education systems and faculties are uploading courseware to student information systems.

**No**	**Conceptual elements**	**Effects**
1	“LIVE Lectures” within the distance and doorstep education system	3.10 /1.5
2	Access the “LIVE subject lesson” within the distance education system with easy access	2.82/ 1.5
3	The records of the “LIVE COURSE” that I could not attend later and do it again	4.1/1.5
4	“REGISTERED SUBJECT OF STUDENTS	3.2/1.4
5	access the “REGISTERED COURSES” within the distance education system to students	3.10/ 1.5
6	“UPLOAD DOCUMENTS COURSES” in the Student Information System	3.60/ 1.4
7	Complete access “COURSE DOCUMENTS” in the Student Information DEPARTMENT	5.9/1.4
8	The quality of sound Audio of the live lessons	2.3/1.3
9	Satisfaction with the VIDEO quality of the system during live classes	2.4/1.4
10	“TECHNICAL SUPPORT” system and a good command of solving problems	2.6/1.4
11	the satisfaction of the work quality and intelligibility	2.6/1.4
12	I am satisfied with the compliance of the Instructors with the system and the “LIVE COURSE” narrations	3.4 /1.4
13	“COURSE DOCUMENTS” in the Student Information System and their content	3.2/1.5
14	“LIVE COURSE PROGRAM” and “COURSE HOURS” within the distance education system	2.7/ 1.5
15	“LIVE COURSES DURATION” within the distance education system	3.4/1.5

Television testimony was likewise a powerful manner to attain college students who do now no longer presently have sufficient assets for online classes.

The time restriction of 1 h (40) 2020; Schleicher and Reimers) other techniques were broadly utilized in Mexico to guide college students in accomplishing home studies and moms who guide homeschooling.

College Students‘ Guidance (41) was completed *via* way of means of government with inside the majority of OECD and associated countries, with the energetic participation of character faculties (39).

### Distance education module

Before the start of the spring semester 2019–2020 pandemic period, the university and all higher education systems uploaded research documents to the research information system. Guides for scientists and students were created and published on the university's website to provide technical information on the handling of distance learning. All courses with links to live courses were edited as distance learning courses according to the 2019–2020 spring semester syllabus, and the taught courses were classified as category registration courses so that students may access the course again.

### Lectures and lecture notes

Researchers have uploaded four live lecture questionnaires and four lecture script questionnaires to the student information system. Participants stated that the instructor was in compliance with the system and was satisfied with the description of the “live course” (a = 3.44) and the duration of the live lesson (a = 3.40).

### Teachers' willingness to support digital learning

**Figure 4:**
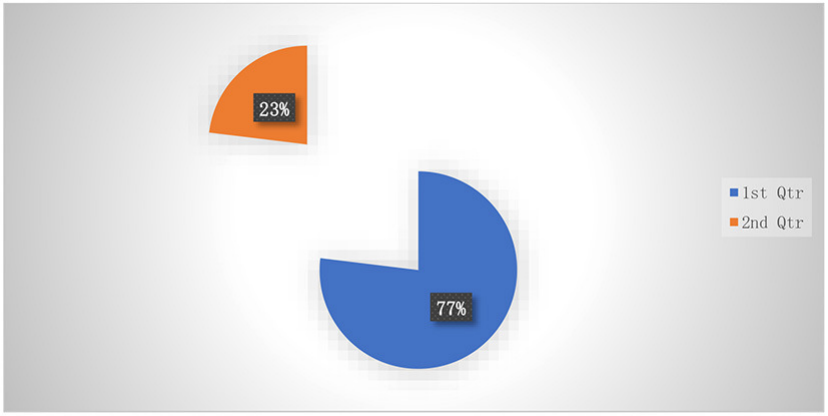
Preparedness to support digital learning.

Figure 4 expressed a sturdy preference for practice withinside the use of records and communique technology. Seventy-seven percent of instructors acquired specialized ICT training, and 23% indicated a want for extra ICT training.

Distance learning has become the lifeblood of education during the pandemic, but the potential of virtual technology extends far beyond the temporary nature of the crisis. Bridging time and space with multiple codecs, this technology Teachers and students to have access to specialized content that goes well-beyond textbooks. An intelligent virtual learning system works with teachers to challenge not only science students' learning styles but also their efforts, hobbies, boredom, or difficulty. In this way, study and work at the same time (42) This structure accommodates the learning experience of healthy college students with incredible granularity and accuracy. In this way, digital labs allow students to design, implement, and investigate experiments instead of conducting preliminary research for undergraduates.

Additionally, the Trainer's ability to contribute acquired expertise and act as a collaborator, coach, mentor, and expert evaluator diminishes over time. According to the 2018 OECD Teaching and Learning International Survey (TALIS), only 53% of teachers allow students to use facts and communications.

Data on international, global and regional public goods, grant commitments from the OECD Creditor Reporting System will be attributed to these categories (about 15% for global public goods and 15% for regional public goods) and other traditional ODA (official development assistance), with the remaining 70%) (43). The best control programmers depend often on stable troubles and curriculum content material substances that contain collaborative and energetic gaining knowledge of talents (41). Total government consumption is also related to the ODA/GDP ratio, which could also be interpreted as the fact that larger governments are associated with stronger altruism. Of the other indicators selected, only public spending on education is significantly correlated with the share of ODA in GDP. By contrast, the combined spending on health, education, and defense is correlated with the share of GPG in total ODA. This supports the hypothesis that the total public spending on education, health, and defense is associated with the relative preference of donors for public goods Trainers, on the alternative hand, are ways much more likely to wait for guides and seminars than different collaborative professionals.

**Figure 5:**
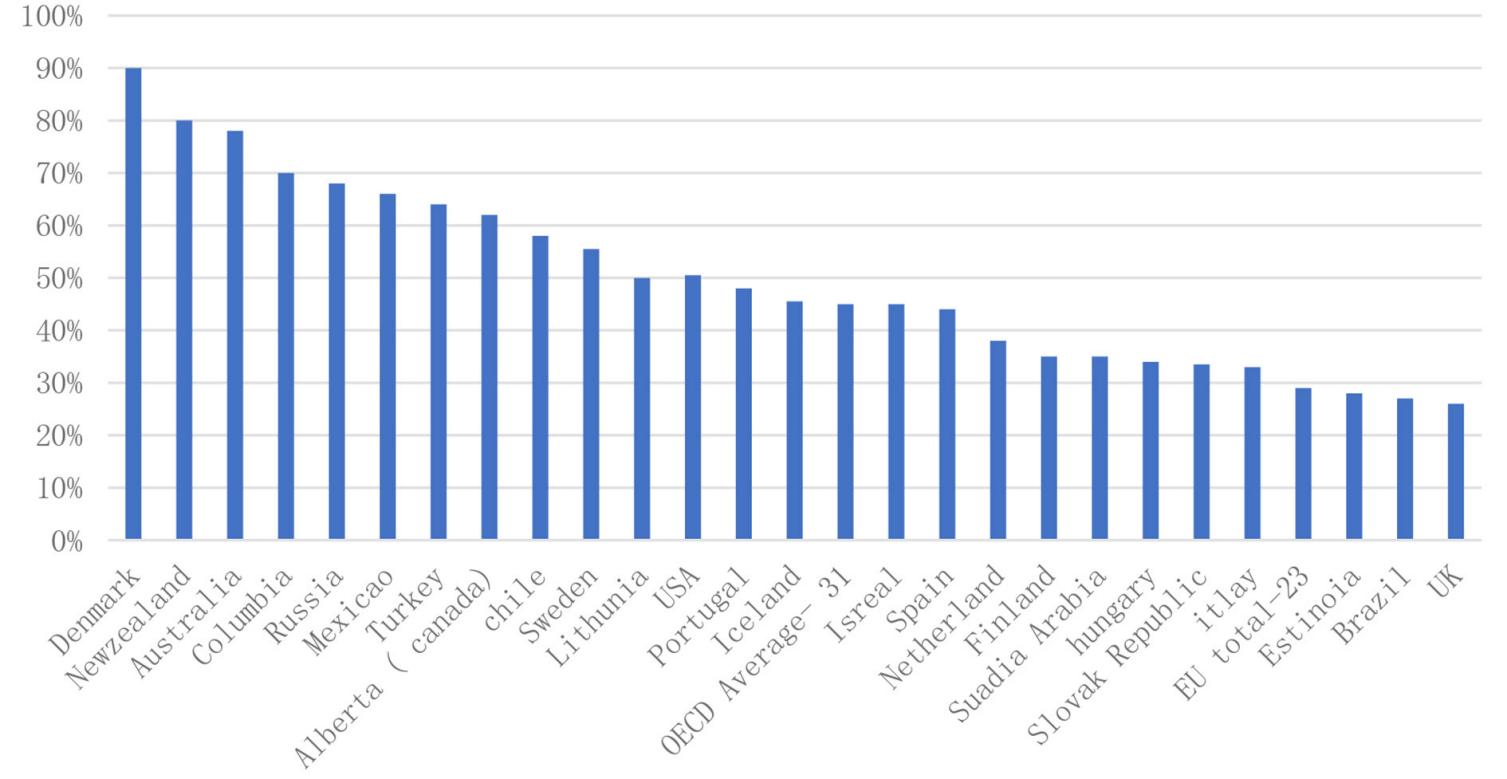
Percentage of secondary education teachers who “frequently” or “always” have students use ICT for projects and classroom tasks.

Across OECD data of countries, 76% of secondary school teachers favor private involvement in guides and seminars, and 44% recognize peering, self-discovery, and being part of the broader university system (Figure 5). Participation of ICT talent is critical, especially given the unusual shift to online education at many international OECD sites during the COVID-19 lockdown. Even before the crisis, the educator made it clear that she preferred ICT-related schooling. This was the second most important school preference after the students' personal educational aspirations, not uncommonly diagnosed as using teachers. On the other hand, the trainer does not simply report her ICT training needs. It also does not rely on distance learning or professional development.

According to professional development records, 36% of a small number of secondary teachers in international OECD countries supported collaboration in an online guide or seminar. Although that was genuine in lots of international locations, there were some exceptions, consisting of Korea and Shanghai (People's Republic of China), in which >90% of teachers pronounced assignment online professional development withinside the preceding year as shown in Table 5.

One of the most effective ways to avoid the COVID-19 epidemic was to keep a social distance. In the faculty context, this means maintaining a safe distance between scientists and staff at 12 m and limiting contact between youth organizations. The level of virus containment achieved determines the term protection in several international locations. For example, in Japan, faculty members must stay within 1 m in low-impact areas (level 1) and faculty members, not in high-impact areas (level 2 or 3). Many countries recommend reducing or halving aspects of education to maintain the required distance between college students (Table 6).

**Table 6:** Shows OECD data of countries and online school percentage by students attended class size and parameters for re-opening of schools.

**Countries**	**SUBJECTS / Seminars attended by the person**	**Coaching with peers and/or introspection as part of a formal school arrangement**	**Online school /Seminar**	**Formal qualification programmer**
Lithuania	97%	70%	48%	20%
Latvia	95%	60%	29%	18%
Slovenia	94%	59%	31%	10%
Australia	93%	70%	71%	11%
Austria	90%	30%	18%	15%
Estonia	88%	51%	38%	11%
Netherland	85%	49%	17%	19%
Belgium	83%	35%	15%	12%
Alberta	83%	40%	40%	9%
New Zealand	80%	78%	33%	11%
Turkey	76%	20%	48%	32%
Russia	77%	77%	70%	10%
Iceland	78%	23%	35%	10%
Czech Republic	78%	55%	47%	15%
Israel	79%	25%	35%	25%
USA	76%	45%	25%	18%
Italy	74%	65%	52%	15%
OECD Average	76%	72%	55%	16%
Chinese Taipei	76%	48%	55%	20%
UK	76%	32%	54%	10%
China	75%	55%	35%	19%
Sweden	78%	18%	24%	8%
Norway	74%	75%	35%	25%

The above-mentioned quick and radical changes in teaching and learning processes have produced significant consequences for students' mental health, i.e., feeling specific emotions and worries. The analysis of the emotions felt by the students showed they were frequently feeling bored, anxious, and frustrated, but also hopeful and joyful, this may be the response and attributed to the fact that the start of the pandemic coincided with the beginning of the 2020 academic year, whereas in the northern hemisphere the academic year was nearing its end, i.e., students in the Southern Hemisphere may be more worried about course delivery and assessment throughout the academic year, rather than just the end of the program. A similar ranking of continents for anxiety was found for frustration as the second-most devastating emotion.

**Figure 6:**
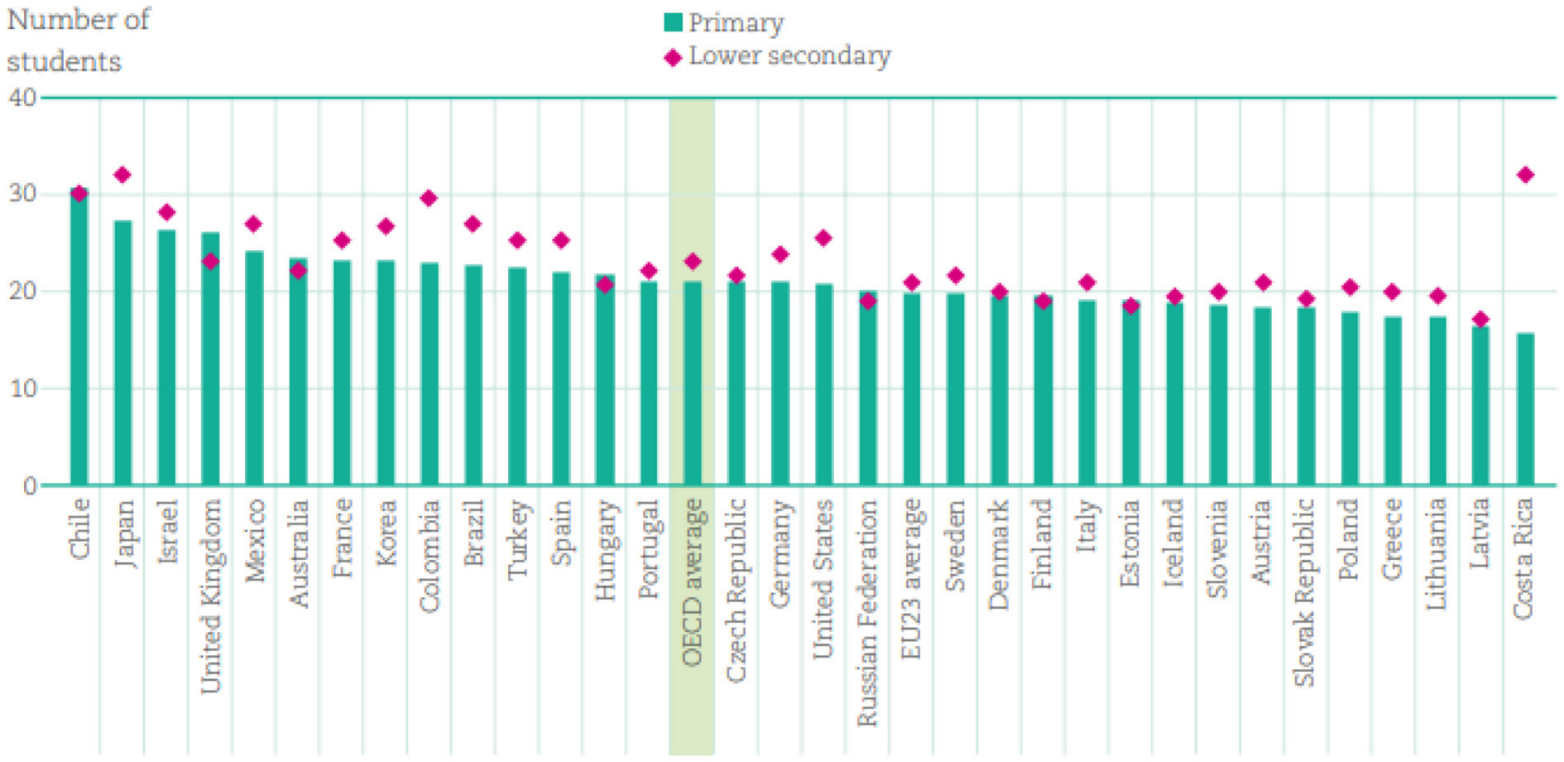
Average class size, by the level of education (2018).

On the other hand, when analyzing positive emotions, North America appeared to be the continent with the most joyful students and Asia with the most hopeful students. To protect students' mental health as effectively as most countries' governments, health professionals, higher education institutions, student organizations, and NGOs should all collaborate intensively on the process of designing timely and efficient psychological and financial support services for students, and maintaining a safe distance between students and teachers depend on several factors, including study room size, availability, and the number of students per class. In countries with small fashionable sizes, sufficient space to safely accommodate college students may make it easier to comply with the new social distance expansion regulations. France and the UK have the same cap on the number of children after first elegance, but the average elegance size of French public schools was 23 college students, compared to the UK with an average post-elegance of 27 college students. This is also getting smaller. Due to the elegance of the education system, there were more than 30 students (Figure 6).

Although most OECD countries require students of the target age group or a certain level of education (excluding sick college students or students with vulnerable or sick circles of their relatives) to return to high school. Attendance was optional in Canada, the Czech Republic, France, and Spain. There were remote classes and online classes for students who want to live at home. These hybrid measures aim to consistently support teacher resumption while optimizing their ability to extend social distance (39).

Finally, this study addresses another donor concern. This means that greater global provision of public goods could lead to lower levels of aid for the poorest countries. However, the empirical analysis above shows the countries' response that the link between recipient countries' per capita GDP and per capita aid levels is already weak. If development aid was weakly linked to the income of recipient countries, it was unlikely that the countries with the lowest incomes were able to systematically reduce the provision of global public goods.

Table 7 shows the results and the limitations are obtained by regressing total aid and her GPG aid expenditure on income and pooling all donors (all variables are measured in her per capita calculation). The GDP per capita of recipient countries was negatively related to ODA/per capita, whereas the GPG-related ODA per capita was positively related, but the income coefficient is not significant in either regression. Of course, it is still possible that certain beneficiaries decided to give another country (perhaps wealthier) more aid in the form of GPG, thus shortening the aid that certain beneficiaries receive. However, this paper did not confirm the substantial displacement of aid in poor countries was caused by the provision of global public goods by ODA.

**Table 7:** Crowding-out effect of GPGs and the dependent variable.

	**ODA per capita**	**GPG per capita**
GDP per capita	−1.99 × 104	1.68 × 105
	2.99 × 10	2.55 × 10

The results of our survey further demonstrate that on the global level, students were quite satisfied with the organization of all three segments of the pedagogical process: Lectures, tutorials/seminars, and mentorships. When comparing the workload before the transition from onsite to online, somewhat less than half of the respondents reported that in the new learning environment their workload had become larger or significantly larger-the biggest increase reported in Oceania and Europe and the smallest in Asia and Africa, both most probably due to the underdeveloped internet network and a lack of computer skills in higher educational unit.

## Conclusion

The purpose of the study was to shed light on the ways the COVID-19 crisis has impacted student life and to design a set of recommendations for policymakers and higher education institutions concerning how students can be supported during the crisis created by the COVID-19 pandemic.

COVID-19 and the educational structure in developing resilient companies are most likely, especially in the mirror of vocational training. Candidates were exposed to a global fitness catastrophe and watched out for class disruptions. This helped to regain the respect of those who expressed opposition during the economic preservation period. The future can be very uncertain. A pandemic, on the other hand, highlights the sensitivities of the crisis and shows how much of the economy was built. The magnitude of the turmoil that was just witnessed includes not only pandemics but also natural, political, financial, and environmental turmoil. As with education and the definition of the school board, we need to develop the ability to respond appropriately, and the economic systems and skills needed to support it.

This study concluded that the distance learning modules, lectures, scripts, and individual assessments of distance learning. All governments intervene in education to maintain, direct, or change the scope of the proposal. Governments need to invest in educational opportunities because there is no guarantee that the market will provide equal access to educational opportunities.

Mental health problems of patients suffering from epidemics, front-line health workers, and the social and psychological impact on society as a whole show striking similarities between our experience with the virus diseases and COVID-19. However, the scale impact of COVID-19, duration, and uncertainty of future developments led to the intensity and its effects hitherto unprecedented in other viral pandemics. So is the rapid spread of COVID-19, the effect of the biology of the virus as evidenced to close connections in international travel and levels of immigration. At the same time, the impact on the global economy shows complex the way humanity is interdependent and connected through its various communities, institutions, and global infrastructure.

## Data availability statement

The original contributions presented in the study are included in the article/supplementary material, further inquiries can be directed to the corresponding author/s.

## Author contributions

FL contributed to the motivation, the interpretation of the methods, the data analysis and results, provided the draft versions and revised versions, references, and provided related concepts, and minor recommendations, and extracted the conclusion and discussion.

## Funding

This article was supported by the Ministry of education of Humanities and Social Science Youth Fund project in 2021 Research on the mechanism, mode and realization path of the deep integration of digitization in the new era and ideological and political education in Colleges and universities (Project No.: 21YJC710034).

## Conflict of interest

The author declares that the research was conducted in the absence of any commercial or financial relationships that could be construed as a potential conflict of interest.

## Publisher's note

All claims expressed in this article are solely those of the authors and do not necessarily represent those of their affiliated organizations, or those of the publisher, the editors and the reviewers. Any product that may be evaluated in this article, or claim that may be made by its manufacturer, is not guaranteed or endorsed by the publisher.
